# More than Just a Monolayer: the Multifaceted Role of Endothelial Cells in the Pathophysiology of Atherosclerosis

**DOI:** 10.1007/s11883-022-01023-9

**Published:** 2022-04-11

**Authors:** Marion Mussbacher, Klaudia Schossleitner, Julia B. Kral-Pointner, Manuel Salzmann, Astrid Schrammel, Johannes A. Schmid

**Affiliations:** 1 Department of Pharmacology and Toxicology, University of Graz, Graz, Austria; 2 Department of Dermatology, Skin and Endothelium Research Division, Medical University of Vienna, Vienna, Austria; 3 Ludwig Boltzmann Institute for Cardiovascular Research, Vienna, Austria; 4 Department of Internal Medicine II/Cardiology, Medical University of Vienna, Vienna, Austria; 5 Institute of Vascular Biology and Thrombosis Research, Medical University Vienna, Schwarzspanierstr. 17, 1090 Vienna, Austria

**Keywords:** Endothelial cells, Atherosclerosis, Endothelial dysfunction

## Abstract

**Purpose of the Review:**

In this review, we summarize current insights into the versatile roles of endothelial cells in atherogenesis.

**Recent Findings:**

The vascular endothelium represents the first barrier that prevents the entry of lipoproteins and leukocytes into the vessel wall, thereby controlling two key events in the pathogenesis of atherosclerosis. Disturbance of endothelial homeostasis increases vascular permeability, inflammation, and cellular trans-differentiation, which not only promotes the build-up of atherosclerotic plaques but is also involved in life-threatening thromboembolic complications such as plaque rupture and erosion. In this review, we focus on recent findings on endothelial lipoprotein transport, inflammation, cellular transitions, and barrier function.

**Summary:**

By using cutting-edge technologies such as single-cell sequencing, epigenetics, and cell fate mapping, novel regulatory mechanisms and endothelial cell phenotypes have been discovered, which have not only challenged established concepts of endothelial activation, but have also led to a different view of the disease.

## Introduction

Endothelial cells (ECs) comprise the innermost layer of all blood vessels and act as gatekeepers of vascular homeostasis by regulating vascular permeability, leukocyte adhesion/extravasation, vascular tone, and hemostasis. Depending on the location and the vascular bed, ECs are exposed to various degrees and types of shear stress, which not only influence their shape, but also affect intracellular signaling and gene expression. Thus, endothelial cells from veins, which are exposed to low blood pressure and flow, differ from those of arteries that are subjected to high pressure and require a robust layer of smooth muscle cells (SMCs) to maintain mechanical stability. By regulating contractility of this smooth muscle layer, arterial ECs adjust local blood flow and contribute to the supply of the underlying tissue with oxygen, nutrients, and transmitters. Apart from venous and arterial vascular beds, ECs are found in capillaries and lymphatic vessels, where they contribute to exchange of fluid and proteins. With respect to the barrier function of the endothelial layer, we distinguish between continuous, fenestrated, and discontinuous endothelium. The first is found in most vascular beds and takes on its extreme in the brain, where the tight junctions of the monolayer form the blood–brain-barrier. Fenestrated endothelium with pores up to 100 nm is found at anatomical sites such as kidney and intestine, where transcellular transport processes or filtration tasks must be fulfilled. Finally, discontinuous endothelium occurs in certain organs like liver or spleen with pores up to 200 nm and additional gaps up to 1 µm, which sometimes lack the basal membrane. Thus, ECs can vary significantly in form and function, while they are also characterized by a high degree of plasticity allowing them to change their phenotype according to local requirements.

The endothelial layer is covered by a dense glycocalyx—a gel-like layer of proteoglycans and extracellular matrix components, which is involved in transendothelial transport, e.g., of lipoproteins, and which can be lost or reduced in inflammation [1].

Under homeostatic conditions, ECs prevent platelet activation, blood clotting, and leukocyte adherence/infiltration by secreting/expressing mediators such as nitric oxide (NO), prostacyclin, tissue plasminogen activator (t-PA), and antithrombin III [2]. However, once activated by inflammatory stimuli, ECs upregulate cellular adhesion molecules such as E-selectin, intercellular adhesion molecule (ICAM), and vascular cell adhesion molecule (VCAM), which trigger leukocyte rolling on the endothelial surface followed by leukocyte diapedesis. Local immune cell activation fuels tissue inflammation, which increases endothelial permeability enabling the entry of plasma proteins to the interstitial space.

Inflammatory activation of endothelial cells and endothelial dysfunction are key events in the initiation of atherosclerosis. This was elegantly demonstrated in a long-term follow-up study demonstrating that patients with endothelial dysfunction have a significant risk for the development of cardiovascular events [3, 4]. In atherosclerosis, disturbed endothelial homeostasis facilitates the permeation and trapping of lipoprotein particles in the subendothelial space, which become oxidatively modified and are sensed as danger-associated molecular pattern (DAMP) by both innate and adaptive immune cells. In addition, some ECs may transdifferentiate into mesenchymal cells (endothelial to mesenchymal transition, EndMT) and promote atherogenesis by loss of cell–cell contacts and deposition of extracellular matrix [5]. Besides initiating immune responses and transdifferentiation processes, modified lipoprotein particles are taken up by macrophages and smooth muscle cells leading to the formation of foam cells and fatty streak build-up. Progressive intracellular lipid overload and curtailed efferocytosis cause cell death and necrotic core formation leading to the thickening of the vascular wall and limiting local blood flow and oxygen supply. Consequently, arterial constrictions cause even larger areas of turbulent or oscillatory blood flow, further contributing to EC activation. Lipid-rich plaques with a thin fibrous cap are prone to plaque rupture, which exposes thrombogenic material to the circulation and initiates life-threatening atherothrombotic occlusion. This detrimental process is accelerated by expression of pro-coagulatory tissue factor (TF) and anti-fibrinolytic plasminogen activator inhibitor (PAI-1) by the luminal endothelium [6]. Alternatively, local endothelial denudation due to endothelial apoptosis may trigger thrombus formation by plaque erosion [7].

**Fig. 1:**
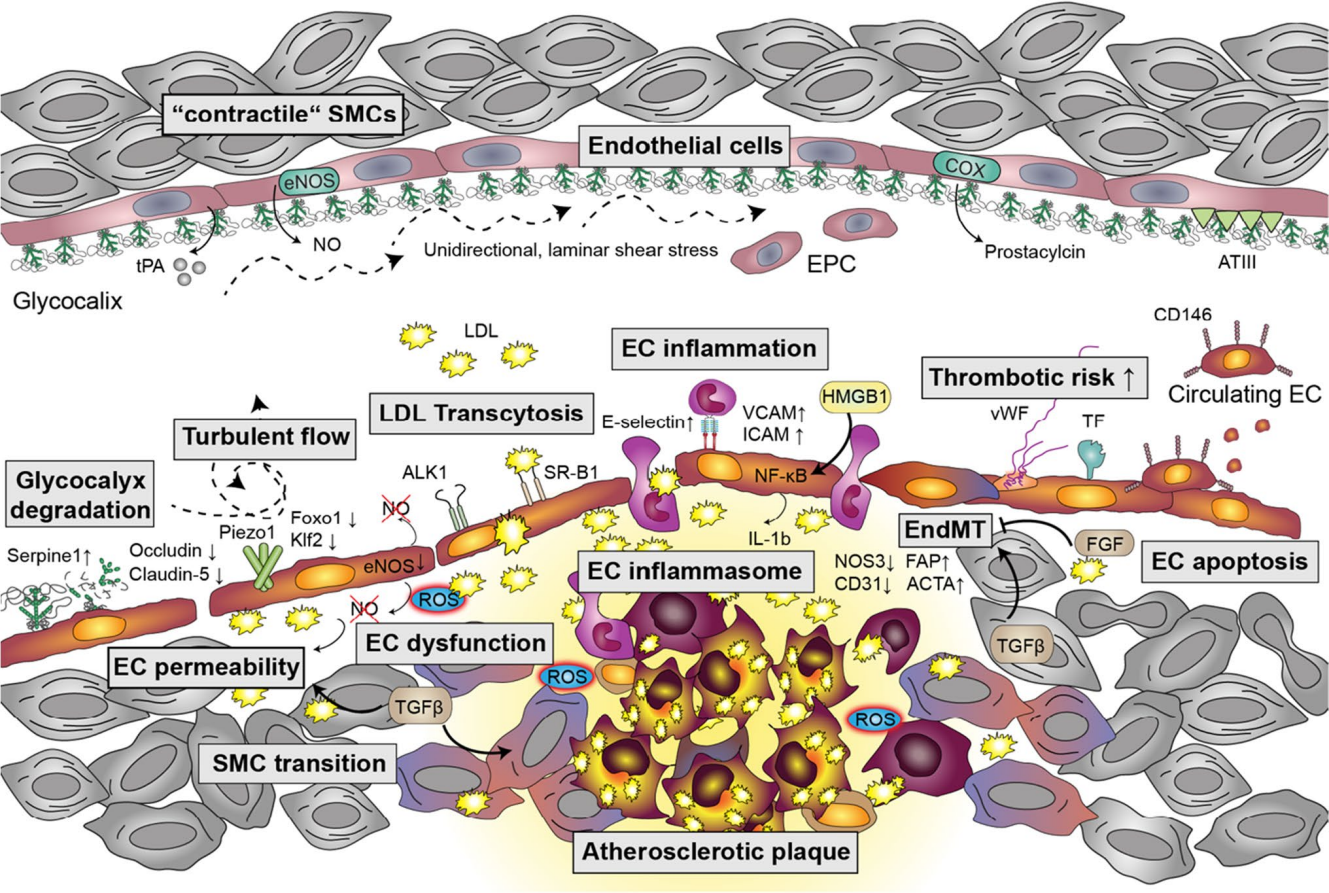
Multifaceted role of endothelial cells in atherogenesis. Under homeostatic conditions (upper part of the scheme), endothelial cells (EC) are covered by a dense glycocalyx and constantly release nitric oxide (NO), which is synthesized by endothelial NO synthase (eNOS). Tissue plasminogen activator (t-PA), antithrombin III (ATIII), and prostacyclin, which is produced by cyclooxygenases (COX), prevent platelet activation and blood clotting. Bone marrow-derived endothelial progenitor cells (EPC) allow EC renewal. Smooth muscle cells (SMCs) are in a “contractile” state, enabling the adjustment of the vascular tone. Under atherosclerotic conditions (lower part of the scheme), the glycocalyx is degraded by plasminogen activator inhibitor (Serpine1) and EC permeability is increased due to downregulation of junctional proteins (e.g., occludin and claudin-5). Mechanosensitive cation channels such as Piezo1 detect turbulent flow and cause the downregulation of Krüppel-like factor 2 (Klf2) and forkhead box O1 (Foxo-1). Endothelial dysfunction is characterized by reduced eNOS expression and decreased NO bioavailability due to elevated levels of reactive oxygen species (ROS). Transcytosis of low-density lipoproteins (LDLs) is mediated by scavenger receptor class B (SR-B1) and potentially activin-like kinase (ALK1). EC inflammation facilitates leukocyte recruitment by increasing the surface expression of E-selectin, vascular cellular adhesion molecule 1 (VCAM), and intercellular adhesion molecule 1 (ICAM). Activation of NF-κB is further enhanced by high mobility group box1 (HMGB1) and initiates the assembly of the EC inflammasome, resulting in the release of interleukin 1β (IL-1β). Endothelial-to-mesenchymal-transition (EndMT) is characterized by decreased expression of CD31 and eNOS/NOS3 as well as increased levels of fibroblast activation protein (FAP) and alpha-actin 2 (ACTA). Whereas transforming growth factor β (TGFβ) promotes EndMT and SMC transition towards a macrophage-like state, fibroblast-like growth factor (FGF) inhibits EndMT. The (athero)thrombotic risk is increased by endothelial expression of tissue factor (TF) and release of von Willebrand factor (vWF). Endothelial apoptosis increases the number of circulating CD146 + ECs in the blood stream

Given the multiple important roles of ECs throughout the initiation and progression of atherosclerosis, understanding molecular mechanisms that regulate EC fate and functions is pivotal for the development of future therapies. This review summarizes recent findings and provides insights into further perspectives (Fig. 1).

### Genetics and Genomics of ECs with an Emphasis on Inflammation

Atherosclerosis is a multifactorial disease associated with various risk factors such as dyslipidemia, diabetes mellitus, smoking, and hypertension. Although heritable factors are assumed to account for over 50% of disease risk [8], knowledge on underlying genetics is limited. Genome-wide association studies (GWAS) provided the first unbiased evidence of hundreds of loci associated with cardiovascular disease and underlying atherosclerosis. Besides well-known lipid metabolism regulating genes such as low-density lipoprotein receptor (LDLR) and proprotein convertase subtilisin/kexin type 9 (PCSK9), genetic variants for inflammatory genes have been identified in GWAS. These include C-X-C motif ligand 12 (CXCL12), SH2B adaptor protein 3 (SH2B3), AB0, human leukocyte antigen (HLA), interleukin 6 receptor (IL-6R), interleukin 5 (IL-5), platelet endothelial cell adhesion molecule 1 (PECAM1), protein C receptor (PROCR), and antisense non-coding RNA in the INK4 locus (ANRIL), whereby SH2B3, PECAM1, and ANRIL are expressed by but not limited to endothelial cells (summarized in [9]). These GWAS studies have recently been extended by single-cell epigenomics of human atherosclerotic lesions, which led to the identification of cell-type specific alterations that predispose for coronary artery disease and myocardial infarction [10]. For ECs, the most prominent changes were seen in chromatin regions that are responsible for cell adhesion, wound healing, and angiogenesis [9]. For a direct comparison of dysregulated EC signatures, another group used blood outgrowth endothelial cells isolated from patients with and without coronary endothelial dysfunction for microarray analysis [11]. Thereby, they identified high mobility group box1 (HMGB1) and laminin gamma1 (LAMC1) to be dysregulated in ECs at an early step of atherogenesis. HMGB1 is sensed as damage-associated molecular pattern (DAMP) and enhances inflammatory responses such as binding of nuclear factor “κ-light chain enhancer” of activated B cells (NF-κB) molecules to DNA [11]. NF-κB is a family of transcription factors closely involved in inflammation and atherogenesis [12]. EC-specific activation of NF-κB via its key kinase IκB kinase 2 (IKK2) significantly accelerates the development of atherosclerosis by increasing leukocyte influx and promoting a phenotypic transition of smooth muscle cells towards a macrophage-like phenotype [13]. Another important mediator of inflammation is the inflammasome, a multiprotein complex composed of a sensor protein and inflammatory caspases, which are in some cases linked by an adapter protein [14]. NF-κB can upregulate various sensor proteins, which bind the adaptor protein apoptosis-associated speck-like protein containing a CARD (ASC), thereby recruiting and activating caspase 1, which converts pro-IL-1β to active IL-1β [15]. The latter is then secreted via gasdermin D-containing pores, activating NF-κB on nearby cells and amplifying the inflammatory circuit. A crucial type of inflammasomes contains NLR family pyrin domain containing 3 (NLRP3), which was first discovered in macrophages as a mechanism for caspase-1 activation and IL-1β processing [16]. However, later it became clear that this fundamental driver of inflammation is also operative in other cells of the vasculature [17]. In endothelial cells, Forkhead box P (Foxp1) has been identified as an important transcription factor repressing the expression of inflammasome components such as NLRP3, caspase 1, and IL-1β, which significantly contribute to the development of atherosclerosis [18]. Using transgenic mice, Zhuang et al. showed that EC-specific overexpression of Foxp1 decreases plaque progression and EC-specific Foxp1 knockout accelerates disease development [18]. Interestingly, FoxP1 was identified to be regulated by Krüppel-like factor 2 (Klf2) and both proteins are decreased in atheroprone vessel regions, which are exposed to disturbed blood flow [18].

Moreover, recent studies identified transforming growth factor-β (TGF-β) as an additional inflammatory driver in atherosclerosis [19•]. In strong contrast to its anti-inflammatory role in smooth muscle cells, endothelial TGF-β signaling promoted vascular permeability and vessel inflammation. Both EC-specific deletion of TGF-β receptors and nanoparticle-targeted suppression of endothelial TGF-β signaling delayed the onset of atheroma formation and reduced leukocyte recruitment and as well as EndMT [19•], underlining the need for additional research into the cell type-specific prerequisites for drug development.

### Cellular Transitions

Novel technologies such as single-cell RNA sequencing (scRNA-seq) revealed the tremendous heterogeneity of endothelial cells as well as other cell types across and within vascular beds with more than 78 EC clusters being identified in 11 murine tissues [20••]. In the murine aorta, three major EC populations have been discovered: (i) CD34^high^ ECs (approx. 56%), which strongly express CD34 and lymphocyte antigen 6a (LY6A); (ii) THY1^high^ ECs that are enriched for Thy-1 cell surface antigen (THY1), transcription box-1 (TBX1), and fms-related tyrosin kinase 4 (FLT4), and “activated ECs,” which expressed genes such as nitric oxide synthetase 3 (NOS3), endothelin 1 (EDN1), angiotensin converting enzyme (ACE), and VCAM1 [21]. Whereas CD34^high^ and THY1^high^ ECs were both associated with endothelial proliferation and vascular development, activated ECs were enriched in pathways associated with vessel dilation and blood pressure regulation [21]. These data were supported by a study from Kalluri and colleagues, which revealed specialization towards lipoprotein handling, angiogenesis, and extracellular matrix production in two of the EC subsets [22]. The heterogeneity of endothelial cells, smooth muscle cells, and immune cells in single-cell datasets from atherosclerosis samples has been summarized in detail [23]. In the murine aorta of ApoE^−/−^ animals on high fat diet (HFD), endothelial cells upregulated classical markers, such as adhesion molecules VCAM-1, ICAM-1, E-selectin, and inflammatory markers such as IL-6, transcription factor hypoxia-inducible factor-1α (HIF1α), but also a plethora of G-protein coupled receptors [24]. Under high-salt, high-fat, or high-plasma glucose diet, an additional EC population with occludin (OCLN)^low^ and plasminogen activator inhibitor 1 (SERPINE1)^high^ characteristics emerged, which pointed to increased EC permeability under conditions of disturbed EC homeostasis as OCLN is associated with tight junction and SERPINE-1 mediates extracellular matrix degradation [21]. In parallel, the “activated EC” cluster was reduced under the same conditions, which coincided with decreased endothelium-induced vasodilation [21].

During plaque growth ECs are exposed to a changing microenvironment which can involve hypoxia, oxidative stress, varying shear stress, and sustained inflammation (e.g., TGFβ). This triggers EC transformation towards states, in which endothelial properties are suppressed and mesenchymal cell behavior is increased. The phenotypic modulation is called EndMT and characterized by decreased expression of endothelial markers such as CD31 and NOS3 and increased levels of mesenchymal markers such as fibroblast activation protein (FAP) and alpha-actin 2 (ACTA) [25]. Moreover, EC-specific lineage-tracing experiments using yellow fluorescent protein (YFP) expressed in an EC-specific manner after tamoxifen-injection revealed that EndMT is common in atherosclerotic lesions of ApoE^−/−^ mice that are fed a HFD and is linked to plaque instability in humans. Expression of FAP + fibroblast-like cells increased in these mice to about 25% of plaque cells and counting of YFP-positive cells suggested that upon 30 weeks of HFD approximately 45% of FAP + cells were derived from endothelial cells [25]. This process seemed to destabilize the plaque via enhanced expression of collagen-degrading matrix metalloproteinases and lower synthesis of collagen [25]. Among eight identified EC clusters obtained from scRNA-seq of aortic and cardiac ECs derived from LDLR^−/−^ mice fed a diabetogenic HFD, three EC clusters expressed mesenchymal marker genes [26]. Further metabolic, transcriptomic analysis of these EndMT clusters revealed diminished fatty acid oxidation, increased inflammation, and extracellular matrix organization [26]. TGFβ and fibroblast growth factor (FGF) signaling cascades are both activated by inflammatory cytokines and oscillatory shear stress. However, they display opposing roles in the induction EndMT: Whereas TGFβ promotes EndMT [19•], endothelial FGF receptor (FGFR) signaling is atheroprotective and knockout of FGFR substrate 2a dramatically increases atherosclerotic plaque burden and neointima formation by extensive EndMT and subsequent fibronectin deposition [27].

Besides ECs, also other cell types are subjected to phenotypic transitions, with vascular SMCs constituting probably the most prominent fraction. It has been shown that inflammation drives SMCs from a contractile to a so-called synthetic state, with higher expression of extracellular matrix components and reduction of contractile elements (for review, see 28). Genetic cell-lineage tracing elucidated that SMCs can further change their identity towards macrophage-like cells in the course of atherosclerosis and it has been postulated that more than 80% of SMC-derived cells lack classical SMC-markers in advanced lesions, while adopting different cellular phenotypes such as macrophage-like, mesenchymal-stem cell-like, or myofibroblastic cells [29]. Recent single-cell genomic analyses revealed the importance of fibromyocytes [30] and also noted that SMCs can adopt an intermediate state between stem cells, ECs, and monocytes (31). Moreover, the inflammatory environment in the plaques also affects the polarization of macrophages derived from infiltrating monocytes, which show a broad spectrum of activation states [32].

Altogether, these findings underline that atherosclerosis leads to a drastically altered environment, which results in significant de-differentiation and trans-differentiation processes of all the different cells of the vasculature.

### Shear Stress

Located at the blood/vessel interface, ECs are constantly exposed to the hemodynamic forces of blood flow, which affect ECs on a transcriptional, epigenetic, and protein level. Whereas unidirectional, laminar shear stress is considered atheroprotective, pulsatile disturbed shear stress such as oscillatory flow that is typically found at arterial branches and vessel wall irregularities induces chronic inflammation [33] and thus enhances atherogenesis. Shear stress is relayed by various mechanosensors such as integrins (e.g., integrinα5—annexin A2 interaction [33]), CD31/PECAM-1, VE-cadherin, and vascular endothelial growth factor receptor 2 (VEGFR2). The interplay between the mechanosensitive cation channel Piezo1, the purinergic P2Y_2_ receptor, and G_q_/G_11_-mediated signaling has been identified to mediate inflammatory signaling and atherosclerosis under disturbed, but not under laminar shear stress [34]. Moreover, plexin D1 (PLXND1) is required for the response of endothelial cells to shear stress and regulates the site-specific distribution of atherosclerosis [35]. However, disturbed flow also induced anti-inflammatory feedback pathways, such as the endothelial adrenomedullin-calcitonin-like receptor (CALCRL), which signals through cAMP and reduces endothelial inflammation and lesion formation [36].

### Endothelial Dysfunction

Endothelial dysfunction (ED) represents an early hallmark of atherosclerosis. It is characterized by a shift of physiological endothelial function towards impaired vasodilation, chronic inflammation, and thrombosis. A key scenario of ED comprises reduced formation and/or bioavailability of the gaseous transmitter NO, which is enzymatically produced by NOS3/eNOS [37]. As a consequence of reduced endothelial NO concentrations, the soluble guanylyl cyclase (sGC)/cGMP/protein kinase G (PKG) cascade in adjacent SMCs is impaired. From the mechanistic viewpoint, different processes have been identified to contribute to ED. They include uncoupling of eNOS (i.e., formation of superoxide at the expense of NO production due to submaximal cofactor concentrations), aberrant eNOS phosphorylation and dephosphorylation, inhibition of eNOS by endogenous methylarginines, and accelerated breakdown of NO due to supraphysiological production of ROS by different enzymatic sources [38]. eNOS function is complexly regulated at levels of transcription, translation, protein maturation, and cofactor assembly. Posttranslational control of eNOS activity by multisite phosphorylation/dephosphorylation is well characterized; however, other protein modifications including S-nitrosylation, acetylation, and glutathionylation have been reported to modulate enzymatic activity (37). In a recent report, Ser 615 has been identified as site of hydroxyl-linked N-acetyl-glucosamine (O-GlcNAc) modification that entails reduced eNOS activity under conditions of glucose dysregulation observed in type-2 diabetes [39].

Moreover, posttranscriptional modulators of gene expression and altered micro RNAs (miRNA) expression patterns have been described in settings of ED, atherosclerosis, hyperglycemia, obesity, and senescence. With respect to endothelial cells, miR-92a has been reported to regulate eNOS mRNA levels via targeting Klf2 [40]. Moreover, vascular expression of miR-221 and miR-222 has been described to be upregulated in initial atherogenic stages and their expression levels have been negatively correlated with eNOS signaling [41]. In addition, miR-195 and miR-532 that have been suggested as biomarkers of thrombosis were found inversely correlated to eNOS expression [42].

In this context, the synergistic interaction between eNOS and silent information regulator 1 (SIRT1) as well as its upstream modulation by miRNAs has gained scientific interest [43]. Belonging to the group of NAD^+^-dependent protein deacetylases, SIRT1 activates eNOS via lysine deacetylation [44]. Moreover, SIRT1 upregulates protein expression of eNOS in a forkhead box O1- (Foxo-1) or KLF2-dependent manner [45]. Conversely, via a positive feedback loop, eNOS-derived NO positively regulates protein expression of SIRT-1. At the transcriptional level, SIRT1 is regulated by a number of miRNAs including miR-34a [46].

Besides miRNAs, two long noncoding RNAs (lncRNAs), termed spliced-transcript endothelial-enriched lncRNA (STEEL) and lncRNA that enhances eNOS expression (LEENE), have been recently described to increase eNOS mRNA expression via KLF2 [47]. Targeting and/or modulation of endothelial miRNAs and lncRNAs that directly or indirectly affect eNOS expression might represent a novel therapeutic approach to counteract ED and associated pathologies.

### Lipoprotein Transport

For decades, passive movement of LDL particles across a compromised endothelial barrier was believed to induce atherogenesis; however, only recently it became clear that LDL can be transported via endothelial transcytosis, which represents an active process that is mediated by several endothelial receptors such as scavenger receptors class B type 1 (SR-B1) [48••] and activin-like kinase 1 (ALK1). Transcytosis of LDL by SR-B1 requires dynamic interaction with dedicator of cytokinesis 4 (DOCK4), which acts as guanine nucleotide exchange factor for Ras-related C3 botulinum toxin substrate 1 (RAC1) and is essential for LDL-SR-B1 internalization. EC-specific knock-out of SR-B1 showed reduced uptake of LDL by artery wall macrophages and severely decreased atherogenesis in the absence of changes in plasma lipid levels or aortic inflammation [48••]. Interestingly, endothelial deletion of the alarmin HMGB1 was reported to downregulate SR-B1 levels via its upstream transcription factor sterol regulatory element-binding protein 2 (SREBP2) [49], which likewise decreased lesion size in EC-specific HMGB1^−/−^ LDLR^−/−^ mice [49], linking inflammation and LDL transcytosis.

By combining a genome-wide RNAi screen of endothelial cells with GWAS data sets, ALK1—a member of the TGF-β receptor superfamily—was identified as additional receptor to mediate endothelial/aortic LDL uptake in vitro and in vivo [50]. However, data on the role of ALK1 in atherosclerosis are still missing and limited by its role in angiogenesis, as loss-of-function mutations in ALK1 cause vascular malformations in humans [51]. Interestingly, both SR-B1 and ALK1 are localized in caveolae, which are specialized membrane microdomains involved in endocytic processes [52]. Absence of caveolin 1—the main protein constituent of caveolae—in LDLR^−/−^ mice reduces LDL transport across the endothelium and decreases vascular inflammation in early-stage atherosclerosis independent of eNOS activity [53, 54]. Although binding of LDL and oxidized LDL (oxLDL) to CD36 and oxLDL receptor 1 (LOX-1) have been demonstrated, none of these receptors affects endothelial LDL transcytosis [49].

In addition to endothelial transcytosis, endothelial breaches and hemorrhage were found in the intima of carotid arteries of ApoE^−/−^ mice at sites of local flow perturbation, which attracts leukocytes and promotes fatty streak formation [55]. Thus, recent findings indicate that fatty streaks can be the result of active transport and retention of oxLDL as well as deposition at sites of injury [55].

### Barrier Function

Activation of endothelial cells results in a reduction of vascular barrier function, increased paracellular and transcellular transport, and, enhanced deposition of fatty streaks in areas of disturbed flow. Pathways that increase endothelial permeability include targets of HIF1-α and NF-κB, such as vascular endothelial growth factors, IL-1β, IL-6, and tumor necrosis factor α (TNFα), but also TGFβ or ROS. In recent years, it has become known that metabolic programs and atherosclerosis are linked by eNOS uncoupling, excess ROS, and the production of advanced glycation products [56]. Additionally, adenosine monophosphate–activated protein kinase (AMPK) can phosphorylate eNOS to decrease NO formation [57] and it can control tight junction protein cingulin to regulate barrier function [58]. Disturbing eNOS can directly and indirectly lead to the disruption of endothelial barriers [59].

Yet, the vascular system harbors mechanisms to counteract these disruptive processes for example by the activation of protective Tie2 signaling through platelets and von Willebrand factor (vWF) [60]. Transcriptional processes downstream of sphingosine-1-phosphate receptor 1 (S1PR1) in aortic endothelial cells showed suppression of the NF-κB pathway and allowed the identification of an endothelial cell subset at aortic branch points [61] that is protected from activation by inflammatory stimuli. Furthermore, recent findings in the field have shown that the barrier function of endothelial cells to small molecules and the transmigration of immune cells are controlled by independent processes. Barrier function towards leak is controlled by tight and adherens junction complexes [62], whereas transmigration is controlled by specific receptor-ligand interactions in combination with substrate stiffness and internal endothelial cell stiffness [63, 64]. Endothelial ICAM-1 and VCAM-1 clusters form cellular protrusions to prepare the endothelial cell for transmigration of leukocytes. The amount of ICAM-1 and VCAM-1 on the cell surface is a response to TNFα or IL-1β signals that determine whether a paracellular or transcellular route is taken for diapedesis [65]. During diapedesis, the paracellular space is sealed by RhoA activation and a contractile actin ring [66]. The combined effects of disruptive and protective programs and the uncoupling of barrier function for small molecules and immune cells will help us understand the formation of atherosclerotic plaques.

### Revascularization and Plaque Stability

It is widely recognized that revascularization of plaques and plaque stability are governed by inflammation and mechanical forces [67, 68]. Meanwhile, many studies have shown that high plasticity of endothelial cells, smooth muscle cells, and fibroblasts plays a major role in the emergence of highly proliferative subsets of cells that form atherosclerotic plaques [22, 69, 70]. Whether these plaques show high vascularization or contain rather more muscle cell layers depends on the origin of proliferative cell clones and the predominant factors that shift transcriptional programs. The number of cells that undergo EndMT is markedly increased during plaque progression and characterizes lesions that are prone to rupture [25]. EndMT results in tenfold less ACTA^+^ myofibroblast-like cells than FAP^+^ fibroblast-like cells. These endothelial-derived fibroblast-like cells were found to produce less collagens and higher levels of matrix metalloproteases and thus could explain plaque rupture. Furthermore, it has been suggested that the rho guanine nucleotide exchange factors SGEF and Trio that control arterial remodeling and endothelial thinning [71] could permit transmigration of immune cells and thereby influence plaque stability. How this knowledge can be translated to favorable clinical outcomes remains to be seen.

### Clinical Perspective and Outlook

Present pharmacotherapies to limit atherosclerosis and associated cardiovascular complications mainly involve lipid-lowering drugs (statins, PCSK9 inhibitors, fibrates), anti-hypertensive drugs (ACE inhibitors, angiotensin II receptor blockers), anti-thrombotic drugs (aspirin), anti-diabetic drugs (metformin), and anti-inflammatory drugs (canakinumab). Although these therapeutic strategies were not designed to target the perturbed endothelium, many of them were shown to directly and indirectly improve endothelial function [72]. For example, statins do not only reduce endogenous cholesterol production by inhibiting hydroxymethylglutaryl-CoA (HMG-CoA) reductase [73], but convey multiple pleiotropic effects. Thus, they improve EC function in patients with ischemic heart failure, which was measured as flow-mediated dilation (FMD) of the brachial artery [74]. Beyond FMD, atorvastatin was shown to elevate levels of circulating endothelial progenitor cells (EPCs). Recent studies highlight circulating EPCs as a promising target to enhance endothelial and vascular recovery by shifting the balance from EC apoptosis to EC regeneration [75]. Similar to the results with atorvastatin, rosuvastatin caused an increase in EPCs in the blood of patients with heart failure. Although rosuvastatin improved FMD, no correlation between FMD and EPC increase was observed [76]. Interestingly, some therapeutic drugs can even cause a reduction of EPCs, as shown in a pilot study with aspirin in patients with atherosclerotic coronary artery disease [75].

Circulating endothelial cells, which can be identified in human blood samples by expression of CD146, have been identified as a marker for EC injury. Patients suffering from ischemic rest pain or acute myocardial infarction show elevated levels of CD146 + cells, which correlate with pro-coagulant vWF and TF. Therefore, elevated circulating endothelial cells might help to distinguish between severe conditions of cardiovascular diseases (e.g., peripheral artery disease with ischemic rest pain of the lower limb and stable arterial diseases) [77].

A potential therapeutic target for the future might be the endothelial glycocalyx, which is essential for endothelial cell function since its breakdown contributes to endothelial lipid accumulation and atherosclerosis. Therefore, maintaining and restoring a functional glycocalyx might be a novel approach to treat atherosclerosis. So far, in vitro data show that albumin stabilizes the glycocalyx and heparan sulfate even repairs the glycocalyx. These interesting findings may find future clinical use [78].

A completely different, but also promising approach, is the therapeutic use of miRNAs, which influence EC functions on a posttranscriptional level. In the clinics, miRNAs have gained center stage as diagnostic markers due to their stable expression. Due to their high diversity, miRNAs possess great potential for therapy; however, miRNA as treatment is still in the early stages of development [79].

Besides their crucial role in disease progression, ECs may also be used as therapeutic tool to increase tolerance of vascular grafts, which are applied to bypass-occluded vessels [80]. To prevent occlusions, grafts are coated with endothelial cells; however, clinical usage of this approach until now is inefficient and very rare. Thus, pre-clinical studies aim to increase vascular function by applying shear stress to improve endothelial retention and thereby reduce graft thrombosis.

In summary, the endothelium fulfills a wide variety of different functions, and we are only beginning to understand its versatile role in the pathophysiology of atherosclerosis. Combining clinical experience with novel technologies such as high-throughput EC profiling including next-generation sequencing approaches and machine learning will open the door for early diagnosis and novel pharmacological therapies.
